# The Indian Basket Trick: a case of delayed paraplegia with complete recovery, caused by misplaced thoracic pedicle screw

**DOI:** 10.1186/s40064-016-2334-y

**Published:** 2016-06-30

**Authors:** Antonin Leroy, Reda Kabbaj, Arnaud Dubory, Manon Bachy, Anne-Isabelle Vermersch, Raphaël Vialle

**Affiliations:** Department of Pediatric Orthopaedics, Armand Trousseau Hospital, Université Pierre et Marie Curie Paris6, 26 Avenue du Dr Arnold Netter, 75571 Paris Cedex 12, France; Department for Innovative Therapies in Musculoskeletal Diseases, Armand Trousseau Hospital, The MAMUTH Hospital-University, 26, Avenue du Docteur Arnold Netter, 75571 Paris Cedex 12, France; Department of Pediatric Neuro-Monitoring, Armand Trousseau Hospital, Université Pierre et Marie Curie Paris6, 26 Avenue du Dr Arnold Netter, 75571 Paris Cedex 12, France

**Keywords:** Misplaced pedicle screw, SEP monitoring, Scoliosis, Late paraplegia

## Abstract

**Introduction:**

Pedicle screw fixation allows purchase of all three spinal columns without encroaching into the spinal canal improving fracture fixation, as well as deformity correction. Fortunately, neurologic injury associated with pedicle screw malposition is rare.

**Case presentation:**

A 19-year-old boy was surgically treated for severe right thoracic scoliosis associated with a Chiari Type 1 malformation and a C6 to T7 syringomyelia. Six months after the initial surgery, the patient was referred to our institution after three weeks of gait disturbances and repeated falls. Imaging showed the gross misplacement of the left T5 pedicle screw, which crossed the center of the vertebral canal. The initial surgery used a freehand technique of pedicle screw insertion, with anteroposterior and lateral postoperative X-ray control. During the surgery, no SEP modifications were noted during pedicle screw placement. However, after insertion of the second rod and scoliosis correction by posterior translation technique, SEP responses decreased considerably. Revision surgery was performed to remove the misplaced screw. During the first three months after screw removal, repeated clinical examinations showed progressive recovery of the neurological deficits. Gait and bladder functions were normal six months after screw removal, and clinical signs of spasticity disappeared. SEP explorations performed at final follow-up showed similar responses to those performed before the initial surgery for scoliosis correction

**Discussion and evaluation:**

Neurologic injury associated with pedicle screw malposition is rare. In early or delayed neurological status worsening, intraoperative or postoperative imaging must be done to detect pedicle screw misplacement. In the current case, thanks to cobalt-chromium and titanium use, MRI and CT scan allowed good visualization of the spinal canal and spinal cord. Experimental studies have shown that neurophysiological monitoring of the spinal cord does not detect moderate compression. In that way, neurophysiological monitoring is an all-or-nothing technique which can misdiagnose early stage of spinal cord injuries. Major penetration of the spinal canal by pedicle screw may conduct to hardware removal.

**Conclusions:**

In early or delayed neurological status worsening, intraoperative or postoperative imaging must be done to detect pedicle screw misplacement. In the current case, thanks to cobalt-chromium and titanium use, MRI and CT scan allowed good visualization of the spinal canal and spinal cord. Major penetration of the spinal canal by pedicle screw may conduct to hardware removal.

## Case report

**Fig. 1:**
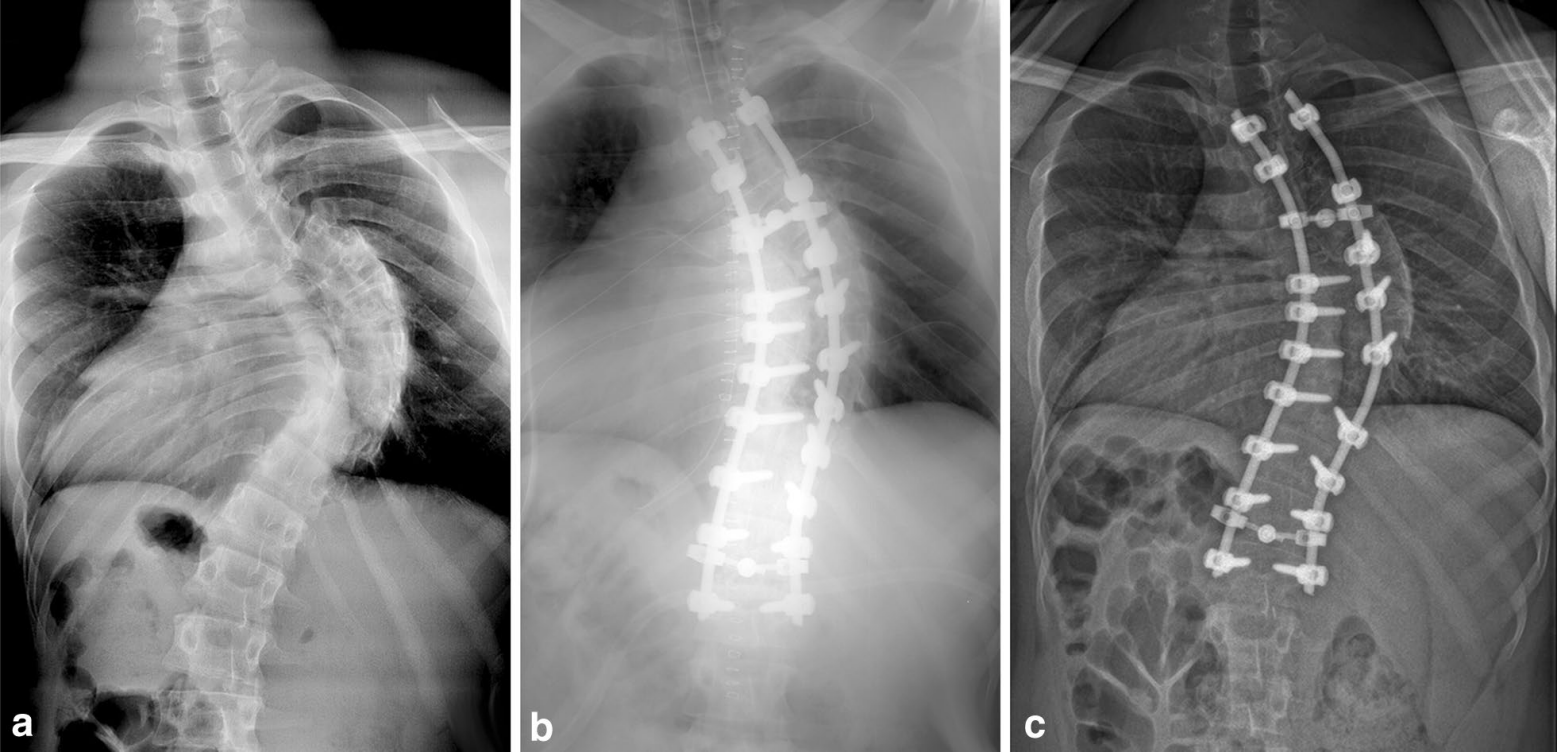
**a** Preoperative frontal X-ray of the right 95° thoracic scoliosis. **b** Postoperative frontal X-ray after index procedure. **c** Postoperative frontal X-ray after revision procedure and screw removal

A 19-year-old boy was surgically treated for severe right thoracic scoliosis associated with a Chiari Type 1 malformation and a C6 to T7 syringomyelia. The Chiari Type 1 malformation was first operated on at the age of 14; the result was considered satisfactory, and the syringomyelia was considered stable between ages 14 and 19. Because Cobb angle increased to 95° (Fig. 1a), the patient received surgical treatment for a T2 to L1 correction, with fusion by posterior approach. The patient underwent posterior spinal fusion and instrumentation, using an all-pedicle screw construct except for the two proximal levels, anchored by hooks. The construct was made using the CD HORIZON^®^ SOLERA™ Spinal System (Medtronic, 710 Medtronic Parkway, Minneapolis, MN 55432-5604, USA) and two 5.5-mm diameter cobalt-chromium rods. The patient was operated on by posterior approach on a Hall frame and with permanent sensory-evoked potential (SEP) monitoring. SEP monitoring protocol consisted in a continuous SEP monitoring with bilateral posterior tibial nerve stimulation and cortical and cervical detection. SEP amplitudes and latencies were recorded in a continuous fashion from patient positioning under general anaesthesia to the end of the surgical procedure.

**Fig. 2:**
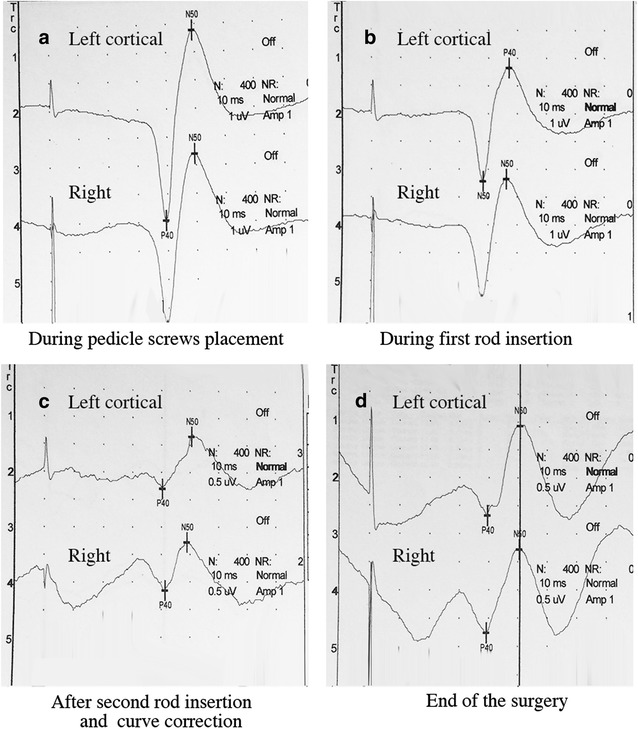
Results of sensitive evoked potentials (SEP) after stimulation of both posterior tibial nerves at various times of the surgery. **a** Results during screws placement, **b** results during first rod insertion, **c** results during second rod insertion and spinal curve correction showing a decrease of cortical amplitudes and increase of latencies, **d** results at the end of the surgical procedure with improved amplitudes and normal latencies

The surgery used a freehand technique of pedicle screw insertion, with anteroposterior and lateral postoperative X-ray control. During the surgery, no SEP modifications were noted during pedicle screw placement. However, after insertion of the second rod and scoliosis correction by posterior translation technique, SEP responses decreased considerably. SEP amplitudes decreased 90 %, while latencies increased significantly (Fig. 2). A subsequent wake-up test showed a complete absence of motor function in both lower limbs. The decision was made to decrease the intensity of correction by contouring the rods (Fig. 1b). This maneuver resulted in improved, but still decreased by 75 % SEP amplitudes and normal (i.e. similar to baseline values) latencies responses, and the patient exhibited no motor or sensory deficit after the procedure. Intraoperative fluoroscopy imaging did not demonstrate any concern for hardware. Daily clinical examinations performed during the first postoperative week showed no motor or sensory deficit. No bowel dysfunction or incontinence were noted during the postoperative course. The details of SEP monitoring values are reported in Table 1.

**Table 1:** Details of SEP monitoring during index procedure

		Screws placement	First rod insertion	Second rod insertion and spinal curve correction	End of the surgery
Left cortical	Amplitude (μV)	6.75	4.16	0.98	1.62
	Latency P40 (ms)	39.4	47.0	40.3	39.4
	Latency N50 (ms)	49.0	38.2	50.4	50.7
Right cortical	Amplitude (μV)	6.01	4.34	0.89	1.51
	Latency P40 (ms)	39.5	38.1	41.2	39.3
	Latency N50 (ms)	49.4	46.2	48.9	50.4

**Fig. 3:**
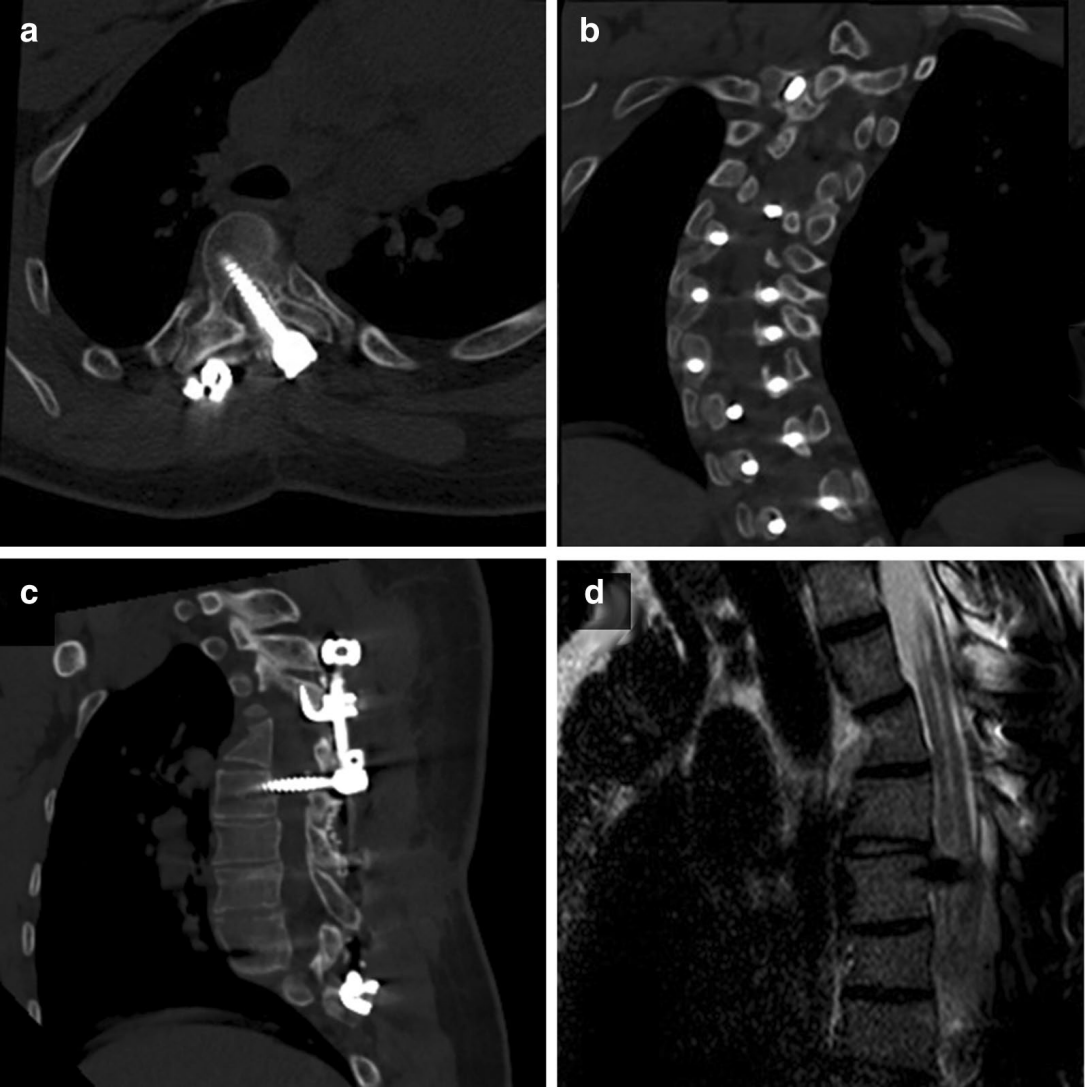
CT scan showing the gross misplacement of the pedicle screw, which crossed the center of the vertebral canal on axial (**a**), coronal (**b**) and sagittal (**c**) planes. **d** Same sagittal plane of spinal MRI showing an increased diameter of the syrinx adjacent to a left T5 pedicle screw crossing the spinal canal

**Fig. 4:**
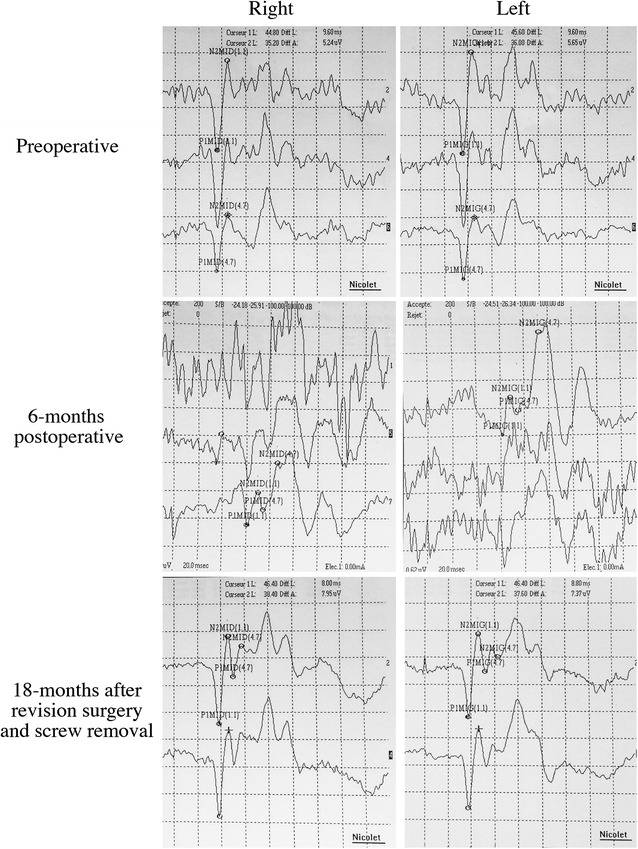
SEP monitoring showing altered responses at 6 months (**b**), with decreased amplitudes and increased latencies compared to preoperative values (**a**). SEP control at final

Six months after the initial surgery, the patient was referred to our institution after 3 weeks of gait disturbances and repeated falls. Clinical examination showed weakness (4/5) of the right psoas, the right flexor digitorum profundus, and the right triceps. There was a right Babinsky sign with increased deep tendon reflexes in both lower limbs. Romberg sign was positive. The patient also presented severe dysuria with bladder distention requiring urinary catheter. There was no recent history trauma, no fever and no remaining biological sign of infection. Axial, sagittal and coronal planes of a thoracolumbar CT scan confirmed the gross misplacement of the pedicle screw, which crossed the center of the vertebral canal (Fig. 3a–c). Spinal MRI was performed to check the C6 to T7 syringomyelia, which showed an increased diameter of the syrinx adjacent to a left T5 pedicle screw crossing the spinal canal (Fig. 3d). SEP monitoring showed altered responses, with decreased amplitudes and increased latencies (Fig. 4).

Revision surgery was performed to remove the misplaced screw. No spinal canal exploration was performed at this time. No cerebrospinal fluid effusion was noted during screw removal, and no meningocele occurred postoperatively. During the first 3 months after screw removal, repeated clinical examinations showed progressive recovery of the neurological deficits. Gait and bladder functions were normal 6 months after screw removal, and clinical signs of spasticity disappeared. The patient reported no motor or sensory deficit at final follow-up, 18 months after screw removal. SEP explorations performed at final follow-up showed similar responses to those performed before the initial surgery for scoliosis correction (Fig. 4), and final MRI confirmed the stability of syringomyelia. No loss of correction was noted after the revision procedure. Final follow-up radiographs showed a partially corrected deformity with good global spinal balance and satisfactory functional results. The patient gave his consent for the use of his personal and medical informations for the publication of this case report and any accompanying images.

## Discussion

Pedicle screw fixation allows purchase of all three spinal columns without encroaching into the spinal canal. This theoretical advantage has been translated to superior clinical results in fracture fixation, as well as in deformity correction (Dickman et al. 1994; Dobbs et al. 2006; Kim et al. 2006; Payer 2005; Yue et al. 2002). For years, introduction of newer techniques, such as fluoroscopically assisted navigation, has lowered pedicle perforation rates (Bransford et al. 2006; Rajasekaran et al. 2007), allowing experienced hands to safely place pedicle screws within the thoracic spine (Hyun et al. 2012; Karapinar et al. 2008; Kim et al. 2004; Vialle et al. 2014; Samdani et al. 2010). Suk et al. (Suk et al. 2001) evaluated more than 4000 thoracic pedicle screws and found a malposition rate of 1.5 %.

Fortunately, neurologic injury associated with pedicle screw malposition is rare. One author evaluated 3204 screws and reported no vascular, neurologic or visceral injuries (Kim et al. 2004). A second author reported a rate of 0.8 % and noted the greatest risk to be on the concave side of the deformity (Suk et al. 2001). This finding is consistent in the literature (Hicks et al. 2010) and suggests that a significant degree of medullary displacement and/or compression is necessary to produce neurophysiological changes (Wadouh et al. 1985; Feng et al. 2014). In early or delayed neurological status worsening, intraoperative or postoperative imaging must be done to detect pedicle screw misplacement. In the current case, thanks to cobalt-chromium and titanium use, MRI and CT scan allowed good visualization of the spinal canal and spinal cord. Major penetration of the spinal canal by pedicle screw may conduct to hardware removal.

## Improving safety

Routine MEP after insertion of each pedicle screw in the thoracic spine, as well as triggered EMG stimulation of each pedicle screw to rule out breaching once all screws have been inserted, could improve safety in challenging cases (Calancie et al. 2014a, b). As in the present case, according to several clinical studies (Danesh-Clough et al. 2001; Raynor et al. 2002; Reidy et al. 2001; Rodriguez-Olaverri et al. 2008; Shi et al. 2003), current intraoperative neurophysiological monitoring techniques do not always accurately reveal the presence of thoracic pedicle screws within the spinal canal. Experimental studies have shown that neurophysiological monitoring of the spinal cord does not detect moderate compression. In that way, neurophysiological monitoring is an all-or-nothing technique which can misdiagnose early stage of spinal cord injuries. Also, a significant delay in identifying the lesion reduces response time to reverse the damage by removing the incorrectly positioned implants (Feng et al. 2014; Flynn and Sakai 2013).

As patient safety becomes more and more of a concern, the use of intraoperative CT scanning, with real CT imaging or CT fluoroscopy of pedicle screws post-insertion, may become a standard of care in the future (Flynn and Sakai 2013).

Some technical tricks, such as the funnel technique (Gaines 2000), allow direct visualization of the entrance into the pedicle, as well as its direction, without the problems mentioned earlier. Two papers have been published evaluating the safety of the funnel technique (Viau et al. 2002; Yingsakmonkol et al. 2002). Because complex spinal deformity distorts anatomic landmarks, we stress the importance of such alternative technique as the “slide technique” as an improvement of the funnel technique (Vialle et al. 2014). The slide technique is a freehand technique for thoracic pedicle screw placement that passes through the decancelled transverse process could be satisfactory and helpful in our clinical experience, especially in severe thoracic spinal deformities. Unfortunately, this technical trick was not used in the current case and might have avoided the initial medial breach.

## Conclusions

This uncommon case of completely misplaced pedicle screw crossing the spinal canal draw the reader’s attention to the risk of pedicle screw placement in severe thoracic curves. In case of early or delayed neurological status worsening, intraoperative or postoperative imaging must be done to detect pedicle screw misplacement. In the current case, thanks to cobalt-chromium and titanium use, MRI and CT scan allowed good visualization of the spinal canal and spinal cord. Major penetration of the spinal canal by pedicle screw may conduct to hardware removal.
